# Circulating interleukin-6 (IL-6) levels are associated with aortic dimensions in genetic aortic conditions

**DOI:** 10.1371/journal.pone.0214084

**Published:** 2019-03-18

**Authors:** Daishi Fujita, Liliana Preiss, Kenichi Aizawa, Federico Asch, Kim Eagle, Toru Suzuki, On behalf of the GenTAC registry investigators

**Affiliations:** 1 Division of Clinical Pharmacology, Department of Pharmacology, Jichi Medical University, Shimotsuke, Tochigi, Japan; 2 Division of Biostatistics and Epidemiology, RTI International, Rockville, Maryland, United States of America; 3 MedStar Health Research Institute, Washington, DC, United States of America; 4 Division of Cardiovascular Medicine, Department of Internal Medicine, University of Michigan, Ann Arbor, Michigan, United States of America; 5 Department of Cardiovascular Sciences, University of Leicester, Leicester, United Kingdom; 6 National Institute for Health Research Leicester Biomedical Research Centre, Leicester, United Kingdom; 7 School of Medicine, Jichi Medical University, Shimotsuke, Tochigi, Japan; IRCCS Policlinico S.Donato, ITALY

## Abstract

**Background:**

Biomarkers that reflect progression of dilatation of the aorta in patients with aortic conditions are needed as surrogate tools to assist in monitoring the condition in a non-invasive manner in combination with imaging procedures. This study aimed to investigate whether biomarkers are associated with aortic dimensions in patients enrolled in the Genetically-Triggered Thoracic Aortic Conditions (GenTAC) registry.

**Methods:**

Plasma samples of 159 patients enrolled in the GenTAC registry were assessed for circulating biomarkers [interleukin-6 (IL-6), matrix metalloproteinase-9 (MMP-9), tissue inhibitor of metalloproteinase-1 (TIMP-1), tissue inhibitor of metalloproteinase-2 (TIMP-2) and transforming growth factor-β1 (TGFβ1)]. Association of circulating biomarker levels with aortic dimensions was investigated.

**Results:**

IL-6 showed significant positive correlations with aortic dimensions at each segment of the aorta, with the correlation increasing in more distal aortic regions (ascending aorta, R = 0.26, p = 0.004; proximal arch, R = 0.35, p<0.0001; transverse arch, R = 0.30, p = 0.0005; mid-descending thoracic aorta, R = 0.40, p<0.0001; thoracoabdominal aorta, R = 0.38, p<0.0001; suprarenal abdominal aorta, R = 0.42, p<0.0001; and infrarenal aorta, R = 0.43, p<0.0001). TIMP-1 showed a significant correlation albeit weaker than IL-6, and also showed increasing correlation towards the distal areas of the aorta.

**Conclusions:**

Circulating IL-6 and TIMP-1 were associated with aortic dimensions in patients with aortopathies enrolled in the GenTAC cohort.

## Introduction

Genetically-triggered aortopathies often result in early manifestation of aortic dilatation. Identification of surrogate biomarkers of aortic dimensions and dilatation would aid in better monitoring patients with aortopathies.

The understanding of molecular underpinnings of genetically-triggered aortopathies has made great progress in the last decade. Molecules such as transforming growth factor-beta (TGF-β) and its downstream intracellular kinase signaling pathway (e.g. mitogen-activated protein kinase [MAPK]/JUN kinase) have been implicated in Marfan aortopathy.[1,2] Inflammatory components such as the cytokine, granulocyte macrophage colony-stimulating factor (GM-CSF), downstream of TGF-β and SMAD3,[3] as well as the IL-6/signal transducer and activator of transcription 3 (STAT3) signaling inflammatory pathway, have also been shown to be involved in regulation of Marfan aortopathy.[4] Further, involvement of extracellular matrix remodeling factors (e.g. matrix metalloproteinase-9, [MMP-9]) has been reported in aortic pathology.[4] At present, our understanding stemming mainly from Marfan aortopathy suggests that a combination of an intrinsic component involving TGF-β, an inflammatory component and an extracellular matrix remodeling component are involved in the underlying multifactorial mechanism of the aortic pathology. These molecules and their downstream pathways involved in aortic pathology provide for potential candidate biomarkers of genetic aortopathies.

The Genetically Triggered Thoracic Aortic Aneurysms and Cardiovascular Conditions (GenTAC) registry sponsored by the National Heart, Lung and Blood Institute (USA) has collected clinical data and biological samples for research purposes from over 3,750 patients with genetically triggered thoracic aortic conditions with aims to promote the understanding and clinical management of aortic and cardiovascular disease with genetic causes.

In the present study, association of circulating biomarkers of aortic dimensions/dilatation in patients enrolled in the GenTAC study was investigated with the aim to identify potential surrogate biomarkers of aortic dimensions/dilatation.

## Methods

### Patient enrollment and sample collection

The registry design and patient enrollment criteria for the GenTAC study have been described elsewhere.[5] All patients submitted written informed consent at the time of participation for the GenTAC registry. The present biomarker analysis was approved by the Bioethics Committee for Epidemiologic Research, Jichi Medical University (approval number Rindai 17-Hen 061). The GenTAC registry contains longitudinal observational data on patients with genetically-induced aortopathies from eight regional clinical centers in the United States. Adult and child patients were enrolled from one or more clinical categories including Marfan syndrome, Turner syndrome, Ehlers-Danlos syndrome, Loeys-Dietz syndrome (LDS), patients with a known genetic mutation (*FBN1*, *TGFBR1*, *TGFBR2*, *ACTA2*, or *MYH11*), bicuspid aortic valve (BAV) with/without known family history of thoracic aortic aneurysm (TAA), bicuspid aortic valve with coarctation, familial TAAs and dissections (FTAAD), Shprintzen-Goldberg syndrome, other aneurysms and dissections of the thoracic aorta under the age of fifty not due to trauma, other congenital heart disease (e.g., tetralogy of Fallot, coarctation), and first-degree family members of probands already enrolled in GenTAC. Clinical data, imaging results, blood and tissue samples were also collected as described.[5]

Patients with available blood samples and aortic imaging data from the registry were enrolled in the present study. Diagnosis, clinical status and imaging data were obtained from registry records.

### Imaging analysis

Computed tomography (CT), echocardiography and magnetic resonance imaging (MRI) images were sent to the GenTAC Imaging Core (iCORE) lab (MedStar Health Research Institute, Washington DC) from each participating institution and were processed using a standardized protocol. Imaging measurement procedures used a unified pre-specified protocol for all imaging modalities to minimize inter-institutional and inter-modality variability.[6,7] In brief, aortic size was measured as the largest cross-sectional dimension in each section of the aorta: sinuses of Valsalva, ascending aorta, proximal arch, transverse arch, mid-descending thoracic aorta, thoraco-abdominal aorta, suprarenal abdominal aorta and infrarenal aorta. Measurements were performed at mid- to end-systole whenever possible using an inner edge to inner edge technique. CT and MRI measurements were performed using a double oblique technique; a true short axis view of the aorta was generated by reformatting a perpendicular view to the long axis of the aorta.[6]

### Biomarker analysis

Plasma samples were stored in ethylenediaminetetraacetic acid (EDTA)-Na after centrifugation at 4°C and then frozen at -80°C. Plasma levels for activated TGF-β1, MMP-9, TIMP-1, TIMP-2 and IL-6 were measured using commercially available quantikine ELISA kits according to the manufacturer’s instructions (ELISA kits from R&D systems with exception of MMP-9 from Omnikine). TGF-β1 samples were first acid-activated with 1N HCL before measurement in duplicate on an automated Zephyr platform (PerkinElmer, Waltham, Massachusetts).

Post-surgical (e.g. aortic, non-aortic within two weeks) and recent dissection samples were excluded given possible confounding effects on biomarker levels.

### Statistical analysis

Data are presented as medians with interquartile range (IQR). The Kruskal-Wallis test was performed for comparison among multiple groups (as the values of measured biomarkers were not normally distributed) and Spearman rank test for correlations between biomarker and aortic dimensions. P<0.05 was considered significant. Statistical Analysis Software/SAS ver 9.04 (SAS Institute, Inc., Cary, North Carolina) was used for statistical analysis.

## Results

### Patient demographics

Two hundred samples with sufficient amount to allow for biomarker measurements (>0.5 ml remaining plasma) were analyzed. Forty-one samples collected within two weeks of aortic dissection or aortic surgery were excluded due to possible confounding effects resulting in a final 159 samples for the present analysis.

Patient demographics of the enrolled 159 patients are shown in Table 1. Patients consisted of BAV with coarctation, BAV with enlargement, vascular Ehlers-Danlos, FTAAD, Loeys-Dietz syndrome, Marfan syndrome, other aneurysms and dissection < 50 years old, other congenital heart diseases, and Turner syndrome. One hundred and twenty-eight patients (80.5%) were diagnosed with annuloaortic ectasia, and 152 patients (96.5%) with thoracic aortic aneurysms. A total of 33 patients (20.8%) had at least one of the following histories of cardiovascular surgery prior to imaging; aortic valve repair or replacement, isolated aortic valve replacement (non-root), aortic root replacement (with valve replacement), valve-sparing aortic root replacement, ascending aortic replacement, aortic arch replacement, mitral valve repair or replacement, descending thoracic aortic replacement, and thoracoabdominal aortic replacement. Blood samples of 2 of the 33 patients with surgery were collected before surgery.

**Table 1:** Patient demographics.

		All patients N = 159
	Age	35.5 (14.9)
	Women (n, %)	77 (48.4)
	Hypertension (n,%)	41 (25.8)
	Diabetes (n,%)	6 (3.8)
**Clinical diagnosis**		
	Bicuspid Aortic Valve with coarctation (n,%)	18 (11.3)
	Bicuspid Aortic Valve with enlargement (n,%)	15 (9.4)
	Ehlers-Danlos vascular (n,%)	15 (9.4)
	Familial Thoracic Aortic Aneurysms and Dissections (n,%)	19 (11.9)
	Loeys-Dietz syndrome (n,%)	18 (11.3)
	Marfan syndrome (n,%)	20 (12.6)
	Other Aneurysm/Dissection <50 y.o. (n,%)	21 (13.2)
	Other Congenital Heart Disease (n,%)	10 (6.3)
	Turner syndrome (n,%)	23 (14.5)
**Medications**		
	Angiotensin Receptor Blocker (ARBs) (n/N, %)	43/148 (29.1)
	ACE-Inhibitor (n/N, %)	19/143 (13.3)
	Beta-blocker (n/N, %)	88/150 (58.7)
	Calcium Channel Blockers (n/N, %)	13/144 (9.0)
	Statins (n/N, %)	30/144 (20.8)
	Other BP lowering drugs (n/N, %)	10/137 (7.3)
**History of cardiovascular surgery**		
	Any surgery (n,%)	33 (20.8)
	Aortic valve repair or replacement (n,%)	5 (3.1)
	Isolated aortic valve replacement (non-root) (n,%)	5 (3.1)
	Aortic root replacement (valve-replacing) (n,%)	15 (9.4)
	Valve-sparing aortic root replacement (n,%)	7 (4.4)
	Ascending aortic replacement (n,%)	11 (6.9)
	Aortic arch replacement (n,%)	5 (3.1)
	Mitral valve repair or replacement (n,%)	4 (2.5)
	Descending thoracic aortic replacement (n,%)	1 (0.7)
	Thoracoabdominal aortic replacement (n,%)	2 (1.3)
**History of aortic dissection**		
	Any dissection (n,%)	21 (13.2)
	Root dissection (n,%)	7 (4.4)
	Ascending aorta dissection (n,%)	8 (5.1)
	Arch dissection (n,%)	7 (4.4)
	Descending aorta dissection (n,%)	12 (7.6)
	Abdominal dissection (n,%)	13 (8.2)
**Aorta dimension of non-grafted segment**		
	Sinus of Valsalva (root) N = 131	4.06 (3.38–4.56)
	Ascending aorta N = 128	3.36 (2.92–3.97)
	Proximal arch N = 131	2.90 (2.50–3.46)
	Transverse arch N = 131	2.60 (2.20–3.01)
	Mid-descending thoracic aorta N = 127	2.40 (1.97–2.76)
	Thoracoabdominal aorta N = 113	2.11 (1.79–2.50)
	Suprarenal abdominal aorta N = 85	2.02 (1.72–2.38)
	Infrarenal aorta N = 86	1.70 (1.42–2.00)

Denominators are given for variables which have missing data. Age is presented as mean±standard deviation (SD) in years, and aorta dimensions of the non-grafted segments are presented as median (interquartile range [IQR]) in centimeters.

Aortic dissection was not reported during follow-up after blood sampling or imaging collection among the 159 patients. Four patients died of other causes.

### Biomarker measurements

Plasma biomarker measurements are shown in Table 2. Circulating IL-6 and TGF-β1 levels were elevated from the reported normal range, and circulating TIMP-1 and TIMP-2 levels remained within the normal range. TGF-β1 showed a trend for higher levels in Loeys-Dietz syndrome, Marfan syndrome, BAV with coarctation, and Turner syndrome (p = 0.08, Kruskal-Wallis analysis). A trend for higher MMP-9 values was seen in Loeys-Dietz syndrome, other congenital heart diseases, other aneurysms and dissection < 50 years-old, and Turner syndrome (p = 0.07, Kruskal-Wallis analysis). However, there was no statistically significant biomarker specific for a specific diagnosis/condition in the study population.

**Table 2:** Circulating biomarker levels according to diagnosis.

	N	IL-6 (pg/ml)		TGF-β1 (ng/ml)		MMP-9 (ng/ml)		TIMP-1 (ng/ml)		TIMP-2 (ng/ml)	
			*P*		*P*		*P*		*P*		*P*
**Overall**	159	1.2		22.4		63.1		94.8		106.6	
		(0.5–3.0)		(9.7–39.8)		(38.2–99.6)		(78.2–112.8)		(89.6–131.8)	
**Diagnosis**											
Bicuspid Aortic Valve with coarctation	18	1.0	0.23	32.9	0.08	51.8	0.07	84.6	0.31	99.8	0.84
		(0.3–3.1)		(14.0–41.4)		(30.9–84.8)		(71.3–100.0)		(95.2–113.9)	
Bicuspid Aortic Valve with enlargement	15	1.8		15.9		54.5		98.8		97.5	
		(1.3–3.2)		(3.5–22.7)		(32.6–66.0)		(88.3–125.8)		(86.4–122.5)	
Ehlers-Danlos vascular	15	1.1		18.6		56.5		105.0		125.2	
		(0.1–2.2)		(11.4–29.2)		(34.1–86.6)		(84.2–119.7)		(89.5–140.9)	
Familial Thoracic Aortic Aneurysms and Dissections	19	1.4		15.6		47.6		102.0		101.6	
		(0.5–3.4)		(5.6–35.7)		(33.1–81.1)		(71.1–156.8)		(89.6–122.7)	
Loeys-Dietz syndrome	18	1.7		36.9		87.0		99.0		114.8	
		(0.7–3.9)		(11.7–44.0)		(45.0–120.2)		(81.0–124.7)		(105.4–146.0)	
Marfan syndrome	20	0.8		31.4		54.8		93.0		95.0	
		(0.2–3.8)		(9.8–48.1)		(26.0–98.8)		(84.7–109.7)		(80.5–120.6)	
Other Aneurysm/Dissection <50 y.o.	21	1.5		17.0		81.4		96.6		113.9	
		(0.6–3.1)		(11.7–33.3)		(54.5–97.7)		(79.1–112.6)		(89.0–140.2)	
Other Congenital Heart Disease	10	2.3		15.3		72.9		116.3		109.7	
		(0.8–4.2)		(7.5–22.8)		(54.2–136.6)		(90.6–165.5)		(98.6–116.0)	
Turner syndrome	23	0.9		34.3		98.0		90.6		105.3	
		(0.3–1.4)		(13.6–43.5)		(47.5–181.0)		(78.6–108.7)		(85.0–137.4)	

Biomarker measurements are presented as median (interquartile range [IQR]). The normal ranges of each biomarker from healthy volunteers according to the manufacturer’s protocol are as follows; TGF-β1 0.9–1.6 ng/ml (platelet-poor EDTA plasma), TIMP-1 44–304 ng/ml, TIMP-2 19–254 ng/ml. IL-6 values of healthy volunteers are usually below the minimal detectable range. The normal range for MMP-9 was not described in the manufacturer’s protocol.

### Elevated circulating IL-6 levels in patients with aortic dissection

In 21 patients with past history of aortic dissection, circulating IL-6 was significantly higher than in patients without dissection (median 2.6 pg/ml IQR 1.8–5.2 vs median 1.1 pg/ml IQR 0.3–2.4, p = 0.001 respectively). The other biomarkers showed no statistical difference between the two groups [TGF-β1; median 14.3 ng/ml (IQR 7.5–37.9) vs median 22.7 ng/ml (IQR 10.4–40.0), p = 0.45; MMP-9, median 53.7 ng/ml (IQR 30.8–74.7) vs median 65.5 ng/ml (IQR 44.8–108.8), p = 0.11; TIMP-1; median 102.3 ng/ml (IQR 86.8–126.0) vs median 94.0 ng/ml (IQR 77.8–111.5), p = 0.11; TIMP-2, median 106.6 ng/ml (IQR 89.7–131.8) vs median 103.3 ng/ml (IQR 84.4–122.3), p = 0.5).

### Correlation between biomarkers and aortic dimensions

Spearman correlation was used to investigate the association of biomarker levels with aortic dimensions of non-grafted segments (Table 3). IL-6, and to a lesser extent, TIMP-1, showed positive correlation with aortic dimensions at each segment of the aorta except for the sinus of Valsalva. Increasing correlation was seen at the distal end of the aorta with an R value of 0.43 (p<0.0001) at the infra-renal aorta for IL-6, and 0.34 (p = 0.001) at the supra-renal aorta for TIMP-1. Decreasing correlation was seen as the segment shifted proximally toward the ascending aorta but still remained statistically significant. TGF-β1 levels showed negative correlation with aortic dimensions of the thoracoabdominal aorta and supra-renal aorta (R = -0.23, p = 0.01; R = -0.26, p = 0.02, respectively). MMP-9 levels showed negative correlation with the ascending aorta and the proximal arch (R = -0.2, p = 0.02; R = -0.2, p = 0.02, respectively).

**Table 3:** Correlation of biomarker against aortic dimension of non-grafted segments.

		IL-6		TGF-β1		MMP-9		TIMP-1		TIMP-2	
	N	R	*P*	R	*P*	R	*P*	R	*P*	R	*P*
Sinus of Valsalva (root)	131	0.12	0.16	-0.06	0.48	-0.13	0.14	0	0.99	-0.2	0.02
Ascending aorta	128	0.26	0.004	-0.11	0.2	-0.2	0.02	0.15	0.08	-0.06	0.5
Proximal arch	131	0.35	<0.0001	-0.17	0.06	-0.2	0.02	0.21	0.02	-0.01	0.9
Transverse arch	131	0.3	0.0005	-0.17	0.05	-0.08	0.3	0.3	0.001	0.03	0.7
Mid-descending thoracic aorta	127	0.4	<0.0001	-0.09	0.3	-0.13	0.13	0.3	0.0003	-0.15	0.09
Thoracoabdominal aorta	113	0.38	<0.0001	-0.23	0.01	-0.08	0.38	0.29	0.002	-0.11	0.24
Suprarenal abdominal aorta	85	0.42	<0.0001	-0.26	0.02	-0.1	0.62	0.34	0.001	-0.09	0.43
Infrarenal aorta	86	0.43	<0.0001	-0.06	0.6	0.02	0.8	0.31	0.003	-0.18	0.1

*P*: P-value from Spearman rank test.

**Fig 1:**
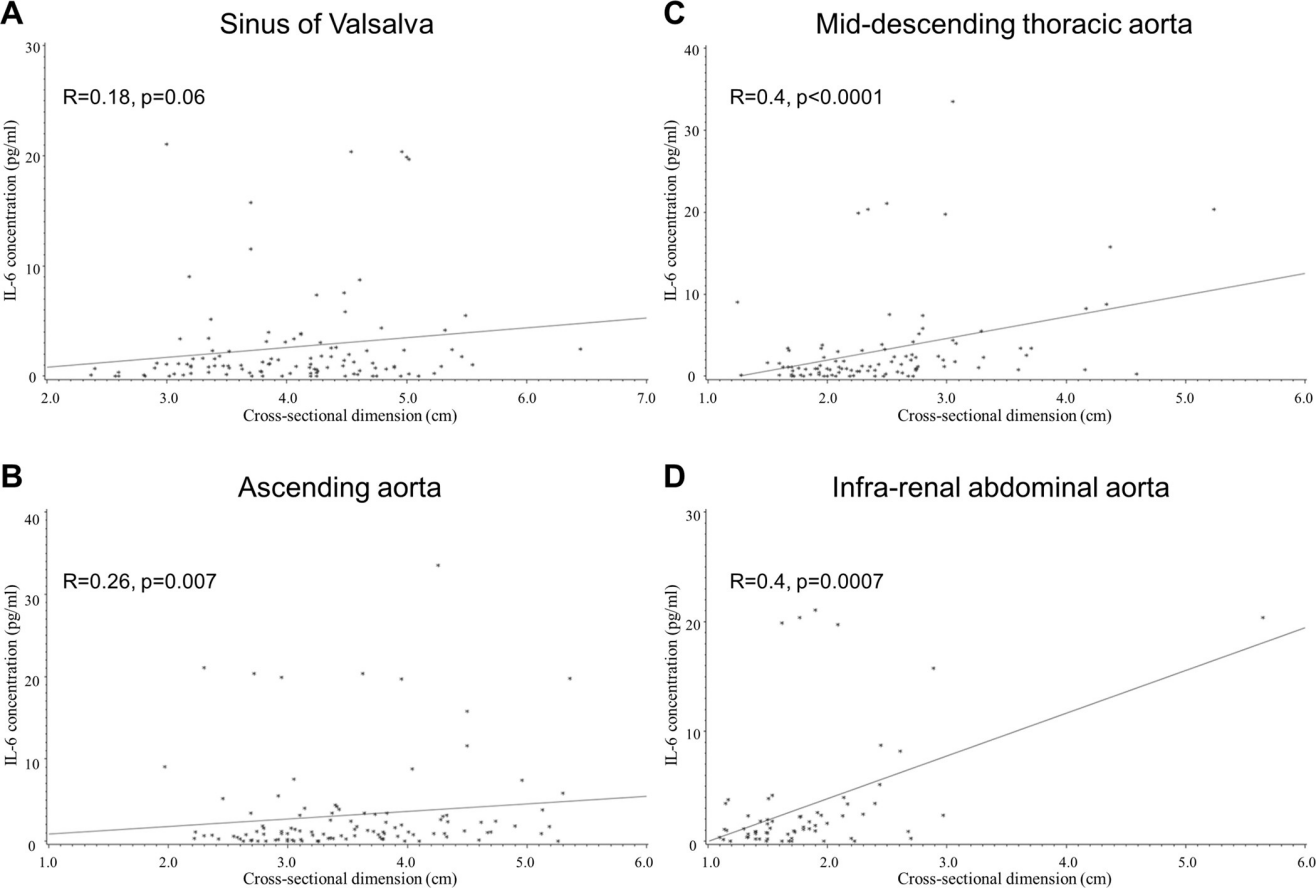
Scatter plot of circulating IL-6 values and aortic dimensions in GenTAC patients without history of aortic surgery. Sinus of Valsalva (A), ascending aorta (B), mid-thoracic descending aorta (C), and infra-renal abdominal aorta (D).

When patients with past history of aortic surgery were excluded to eliminate potential confounding effects, patients without prior aortic surgery showed statistically significant correlations at each segment of the aorta between IL-6 or TIMP-1 and dimensions of the native aorta (Table 4, Fig 1). By contrast, in patients with prior aortic surgery, correlation was not observed between the biomarkers and aortic dimensions. Positive correlation between aortic dimensions and IL-6 or TIMP-1 was seen even after excluding patients with past aortic dissection (Table 4), suggesting that associations between aortic dimensions and IL-6 or TIMP-1 are to some extent independent of former dissection or surgical manipulation. In this group, the infrarenal aorta did not show significant correlation (R = 0.25, p = 0.06 for IL-6, R = 0.23, p = 0.09 for TIMP-1), but correlation remained significant from the ascending to suprarenal aorta. Correlation was observed between IL-6 and TIMP-1 (R = 0.33, p<0.0001), but not among the other biomarkers.

**Table 4:** Correlation of IL-6/TIMP-1 and aortic dimension (non-grafted segment) with or without aortic surgery, aortic dissection.

	All patients			No history of aortic surgery			History of aortic surgery			No history of aortic surgery/dissection		
	N	R	*P*	N	R	*P*	N	R	*P*	N	R	*P*
**Correlation with IL-6**												
Sinus of Valsalva (root)	131	0.12	0.16	113	0.18	0.06	18	-0.2	0.5	103	0.17	0.07
Ascending aorta	128	0.26	0.004	109	0.26	0.007	19	0.39	0.1	98	0.3	0.003
Proximal arch	131	0.35	<0.0001	104	0.36	0.0001	27	0.2	0.32	92	0.34	0.001
Transverse arch	131	0.3	0.0005	101	0.3	0.001	30	0.14	0.4	89	0.28	0.007
Mid-descending thoracic aorta	127	0.4	<0.0001	101	0.4	<0.0001	26	0.25	0.21	89	0.34	0.001
Thoracoabdominal aorta	113	0.38	<0.0001	87	0.37	0.0004	26	0.37	0.06	74	0.3	0.01
Suprarenal abdominal aorta	85	0.42	<0.0001	66	0.43	0.0003	19	0.26	0.28	53	0.36	0.009
Infrarenal aorta	86	0.43	<0.0001	70	0.4	0.0007	16	0.57	0.02	58	0.25	0.06
**Correlation with TIMP-1**												
Sinus of Valsalva (root)	131	0	0.99	113	0	0.99	18	-0.06	0.81	103	0	0.94
Ascending aorta	128	0.15	0.08	109	0.21	0.03	19	-0.24	0.31	98	0.24	0.02
Proximal arch	131	0.21	0.02	104	0.24	0.01	27	0.1	0.6	92	0.24	0.02
Transverse arch	131	0.3	0.001	101	0.25	0.01	30	0.34	0.07	89	0.24	0.03
Mid-descending thoracic aorta	127	0.3	0.0003	101	0.33	0.0006	26	0.1	0.6	89	0.35	0.0009
Thoracoabdominal aorta	113	0.29	0.002	87	0.27	0.01	26	0.28	0.16	74	0.3	0.01
Suprarenal abdominal aorta	85	0.34	0.001	66	0.3	0.007	19	0.4	0.08	53	0.38	0.005
Infrarenal aorta	86	0.31	0.003	70	0.29	0.01	16	0.37	0.1	58	0.23	0.09

*P*: P-value from Spearman rank test

## Discussion

The present study investigated associations of circulating biomarker levels with aortic dimensions in patients enrolled in the GenTAC study. Circulating IL-6 and to a lesser extent TIMP-1 showed modest associations with aortic dimensions.

Investigation of surrogate biomarkers of diagnosis or disease progression in patients with genetic aortopathies including Marfan syndrome has mainly focused on TGF-β1 or MMPs. While Marfan patients, especially those with aortic root dilatation, have been reported to show higher circulating TGF-β1 levels,[8,9] TGF-β1 did not exhibit correlation with aortic dimensions in the present study. Elevated total serum TGF-β1 levels have also been reported in LDS, BAV and FTAAD patients but to not be correlated with aortic dimensions, which is consistent with the present results.[10] Elevated MMP-9 activity has also been demonstrated in the aortic wall of thoracic aortic aneurysms in a murine model of Marfan syndrome,[11–13] and elevated MMP-3 and MMP-9 activities in the circulation of post-surgical samples from Marfan patients as assessed by RT-PCR of peripheral blood mononuclear cells.[14] However, circulating MMP-9 activity did not show association with aortic dimensions in non-operated GenTAC patients in the present study.

Together with other preceding studies,[4,15] the present study sheds light on IL-6 as a biomarker associated with aortic dimensions and to be potentially associated with pathogenesis of genetic aortopathies. Inflammation contributes to aortic pathology of atherosclerotic/aging origin; the present findings extend this concept to contribution of inflammation to aortic disease of non-atherosclerotic/genetic origin as well. Unexpectedly, TIMP-1 was also associated with aortic dimensions in our study. TIMP-1 is a preferential inhibitor for MMP-9, and an elevated MMP-9/TIMP-1 ratio has been reported in the tissue of the aortic wall from patients with thoracic aortic aneurysms.[11–13,16] Upregulation of inflammation in the aortic wall of Marfan mice with concomitant increase in TIMP-1 in the aortic wall has also been recently reported.[17] Our results showed TIMP-1 but not MMP-9 to be associated with aortic dimensions which may be attributable to inflammatory contribution rather than aortic wall remodeling. Correlation between IL-6 and TIMP-1 levels as observed in our study suggests that TIMP-1 is associated with inflammation but this finding needs to be addressed in further research.

### Mechanistic implications

Mechanistically, the described findings are in line with experimental studies that have shown involvement of IL-6 in aortic dilatation. Hypomorphic fibrillin-deficient mice as an animal model of Marfan syndrome which develop spontaneous aortic dilatation and aneurysms of the ascending aorta by three months of age showed increased IL-6 transcription and secretion in aortic tissue at the end of the study at three months, accompanied by increase in secretion of monocyte chemotactic protein-1 (MCP-1) and GM-CSF,[4]. Progressive aortic dilatation was observed when both fibrillin-1 and IL-6 were deficient. In another study, increased blood pressure was shown to induce IL-6 and MCP-1 through phosphorylation of STAT3 in isolated vascular smooth muscle cells, which led to macrophage accumulation in aortic media and aneurysm formation in a mouse model of abdominal aortic aneurysm formation.[18] Although the findings in murine models of aortopathy are not directly applicable to patients given differences in aortic pathology in mice and men,[19] our study complements these previous animal studies to support a role for involvement of the inflammatory cytokine, IL-6, in aortic dilatation in patients with genetic aortopathy.

In a recent study investigating patients with inflammatory aortic aneurysms, significantly elevated serum IL-6 levels were seen in patients with IgG4-related aortic aneurysms. [20] IL-6 levels correlated with adventitial thickness, and IL-6 immunopositive cells and mRNA expression were localized in the adventitia, suggesting that IL-6 in the adventitia is involved in the pathogenesis and progression of aneurysmal formation. Abdominal aortic aneurysms of non IgG4 origin or of atherosclerotic origin also showed increased serum IL-6 compared to normal controls. As our study population was younger with smaller percentage of patients with hypertension, it is not clear whether the above mechanisms are applicable for aortopathies of genetic origin in patients. However, correlation between IL-6 and lesions of the abdominal aorta seems to be consistent among different studies. While the murine experiments also showed that activity of MMP-9 is increased in Marfan model mice, the present study showed TIMP-1 and not MMP-9 to be associated with aortic dimensions. TGF-β1 showed elevated levels in GenTAC patients but an association with aortic dimensions was not observed. Further investigations are necessary to determine the role of matrix metalloproteinase and tissue inhibitor in genetic aortic pathology.

### Clinical implications

The present study shows that circulating IL-6 and TIMP-1 are potential biomarker candidates for monitoring aortopathies (e.g. dilatation) in patients with genetic background. The cross-sectional design and associative nature of the present study does not allow for determination of a causal relationship between biomarkers and aortic dimensions, however if increased biomarker levels are shown to be associated with progressive aortic dilatation in further studies, then this would potentially allow use of biomarkers to monitor aortopathies in patients with genetic conditions in the future. Circulating biomarkers coupled with imaging analysis is envisaged to optimize timing and use of the latter when translated to the clinic.

The normal range for the investigated biomarkers shows large variation among previous reports. Plasma IL-6 levels among normal patients have been reported to show a range from 0.014 to 6.7 pg/ml,[21] and plasma TGF-β1 levels have been reported to show median reference levels of 6.2 (range 1.0–33.1) ng/ml in healthy control subjects.[22] Plasma MMP-9, TIMP-1 and TIMP-2 levels have been reported to be 107.7±53.96 ng/ml, 250.03±53.96 ng/ml, and 58.79±8.26 ng/ml (mean±SD), respectively.[23] It is noted that these normal reference values are derived from control groups higher in age than our study population. The biomarkers investigated in our study all show levels that would fall within their normal reference values but also illustrates that there is much variation in reference values (e.g. different populations and measurement methods).

### Study limitations

The present study was a cross-sectional study, and whether higher IL-6 (and TIMP-1) plasma levels are causative of aortic dilatation cannot be determined from the associative nature of the present study. Although 159 GenTAC patients were enrolled, each subgroup consisted of less than 20 patients each which were insufficient to generate statistically powered disease-specific analyses. There was variation in the interval between imaging and sample collection as well. Standardization of measurements (e.g. imaging) is recommended in guidelines.[24] Absence of a control group also limited identification of a cut-off level. Due to the limited sample amount, some previously described markers associated with aortic pathology (e.g. white blood cell count, fibrinogen, D-dimer, troponin T, N-terminal pro Brain type Natriuretic Peptide[NT-proBNP] and high-sensitivity C-reactive protein) could not be investigated.[25]

## Conclusion

Circulating IL-6 and TIMP-1 were associated with aortic dimensions in patients with aortopathies in the GenTAC cohort. Further research is necessary to clinically translate these biomarkers as surrogate tools for monitoring aortic dimensions and dilatation in patients with genetic aortopathies.

## Supporting information

S1 Table Data set. (XLS)
